# pTx‐Pulseq in hybrid sequences: Accessible and advanced hybrid open‐source MRI sequences on Philips scanners

**DOI:** 10.1002/mrm.30601

**Published:** 2025-07-03

**Authors:** Thomas H. M. Roos, Edwin Versteeg, Mark Gosselink, Hans Hoogduin, Kyung Min Nam, Nicolas Boulant, Vincent Gras, Franck Mauconduit, Dennis W. J. Klomp, Jeroen C. W. Siero, Jannie P. Wijnen

**Affiliations:** ^1^ Department of Radiology and Oncology, Center for Image Sciences University Medical Center Utrecht Utrecht the Netherlands; ^2^ University of Paris‐Saclay, CEA, CNRS, NeuroSpin, BAOBAB Gif sur Yvette France; ^3^ Spinoza Center for Neuroimaging Amsterdam Amsterdam the Netherlands

**Keywords:** hybrid sequence, open source, parallel transmit (pTx), Pulseq, universal pulse (UP), vendor‐neutral

## Abstract

**Purpose:**

To enhance the accessibility of advanced pulse sequences, or parts thereof, through the open‐source Pulseq framework. This work extends the Pulseq framework to Philips MRI systems and incorporates dynamic parallel‐transmit (pTx) capabilities within the constraints of the existing Pulseq format. This enables the hybrid use of Pulseq sequences within the native vendor's scans, leveraging the combined strengths of both approaches. We showcase a new possibility of these techniques: the use of a portable cross‐vendor universal pulse (UP) excitation in a native scan.

**Methods:**

pTx‐Pulseq was implemented to add full dynamic pTx within the Pulseq specification. We developed a Pulseq interpreter for Philips systems, supporting both Pulseq‐only and hybrid sequences. The hybrid mode was used to integrate a UP from the PASTeUR package into a vendor's native scan. Simulation experiments, safety validations, field measurements, and imaging experiments in phantom and in vivo were conducted to verify the interpreter's and UP's functionality.

**Results:**

The interpreter executed Pulseq sequences on a Philips 7T system, accurately reproducing gradient waveforms and dynamic pTx sequences. The real‐time safety systems operated correctly. Phantom and in vivo scans demonstrated comparable image quality to native sequences, validating the effectiveness of the interpreter and the successful cross‐vendor use of universal pulses.

**Conclusion:**

The successful cross‐vendor application of a universal pulse through pTx‐Pulseq in a hybrid sequence demonstrates how advanced MRI techniques can be made accessible. This not only highlights the flexibility and extensibility of Pulseq but also sets the stage for rapid clinical translation of innovative imaging techniques.

## 1 INTRODUCTION

Traditionally, MRI sequences are developed and implemented in vendor‐specific environments. This makes it challenging for researchers to write and compile new sequences, as the necessary access to the vendor‐specific source code can be difficult. Furthermore, it complicates the interchangeability, standardization, and reproducibility of MRI sequences between researchers using different software versions, especially across various platforms from different MRI vendors. Moreover, while specific sequences may benefit research, they may only sometimes become available as a product brought to market by MRI vendors. Those sequences can, therefore, remain confined to individual labs instead of being easily distributed among other institutes, hindering scientific progress.

Various open‐source frameworks have emerged to overcome these barriers, some focusing on data reconstruction or image processing and others designed specifically for pulse‐sequence programming. Frameworks such as Pulseq, Toppe, and gammaSTAR enable hardware‐independent and software‐independent sequence development in high‐level languages such as *MATLAB* or *Python*.^1^, ^2^, ^3^, ^4^, ^5^, ^6^ These sequences can subsequently be imported into other (simulation) software or executed directly on MRI scanners. Pulseq has seen increasing adoption within the MRI research community. Consequently, advanced sequences have been developed in Pulseq and made available through GitHub, such as “Pulseq‐CEST” for chemical exchange saturation transfer MRI and “PulseqDiffusion” for diffusion MRI.^7^, ^8^

Pulseq has previously been supported by four MRI vendors (Siemens, Bruker, GE, and United Imaging) that allow for either high‐level translation of Pulseq into their native MRI sequence format or internally function similar enough to make an interpreter possible.^1^, ^2^, ^9^ Philips MRI scanners, however, use a distinct platform that leverages the repetitive nature of pulse sequences, which historically limited direct Pulseq integration. Nonetheless, recent advancements in Philips' digital hardware capabilities suggest that extensive runtime modifications are feasible, enabling the implementation of a Pulseq interpreter. As Philips supplies a significant portion of the world's MRI scanners, it is worthwhile to make the Pulseq available to this vendor as well.

Moreover, Pulseq to date does not support all features that are supported by the native vendor‐specific environments, such as the control over multiple independent transmit channels. At high field strengths, these parallel transmit (pTx) systems with multiple independent transmit channels can alleviate the inhomogeneous ${\tilde{B}}_{1}^{\left(+\right)}$ and the resulting undesired contrast and signal‐to‐noise ratio (SNR) variations.^10^ pTx can be used for static ${\tilde{B}}_{1}^{\left(+\right)}$ shimming, which uses a fixed set of optimized phases, or for dynamic pTx pulses that use per‐channel waveforms. Dynamic pTx pulses, such as a kT‐points pulse, can be designed for a specific subject to homogenize the effective flip angle across the object at the cost of additional preparation scans and computation time.^11^ Universal pulses (UPs) are optimized over a group and remove the burden associated with subject‐specific pulses.^12^ Previous work has shown that they are transferable between different sites with the same transmit coil setup.^13^ The PASTeUR package of pulses has been shared with over 30 sites of the same vendor,^13^, ^14^, ^15^ although, thus far, not between different MR scanner platforms. We hypothesize that, with a novel conversion process to handle opposing $B_{0}$ directions (detailed in the Appendix A), UPs can be transferred across different platforms using pTx‐Pulseq.

In this work we aim to extend the accessibility of advanced pulse sequences offered by Pulseq. We present the first Pulseq interpreter for Philips MR systems and two extensions to the general Pulseq framework: pTx‐Pulseq and hybrid sequences. pTx‐Pulseq enables both static and dynamic pTx within the constraints of the current Pulseq specification. Hybrid sequences combine or merge Pulseq sequence blocks with vendor‐optimized sequence components. This allows for rapid prototyping of, for example, prepulses or different excitations without the need to replicate the other vendor's optimized sequence components. To demonstrate the effect of these developments, we show how a universal pulse from the PASTeUR package can be converted to pTx‐Pulseq and deployed within a hybrid sequence on a Philips 7T system. This showcases, for the first time, that pTx pulses—once restricted to specific platforms—can now be shared universally through an open‐source framework across different platforms and vendors.

## 2 METHODS

### 2.1 pTx‐Pulseq

#### 2.1.1 Storage of pTx waveforms

The current Pulseq specification (v1.5.0) supports single‐channel radiofrequency (RF) pulses and does not explicitly expose any features offered by pTx systems.^15^ Previous work extended Pulseq with static pTx by introducing new entry fields and consequently altering the file format.^14^ Our proposed pTx‐Pulseq method offers full pTx capabilities with channel‐independent waveforms while aiming to be as backwards compatible as possible. This is achieved without introducing new entry fields and adhering to the current format specification.

**FIGURE 1:**
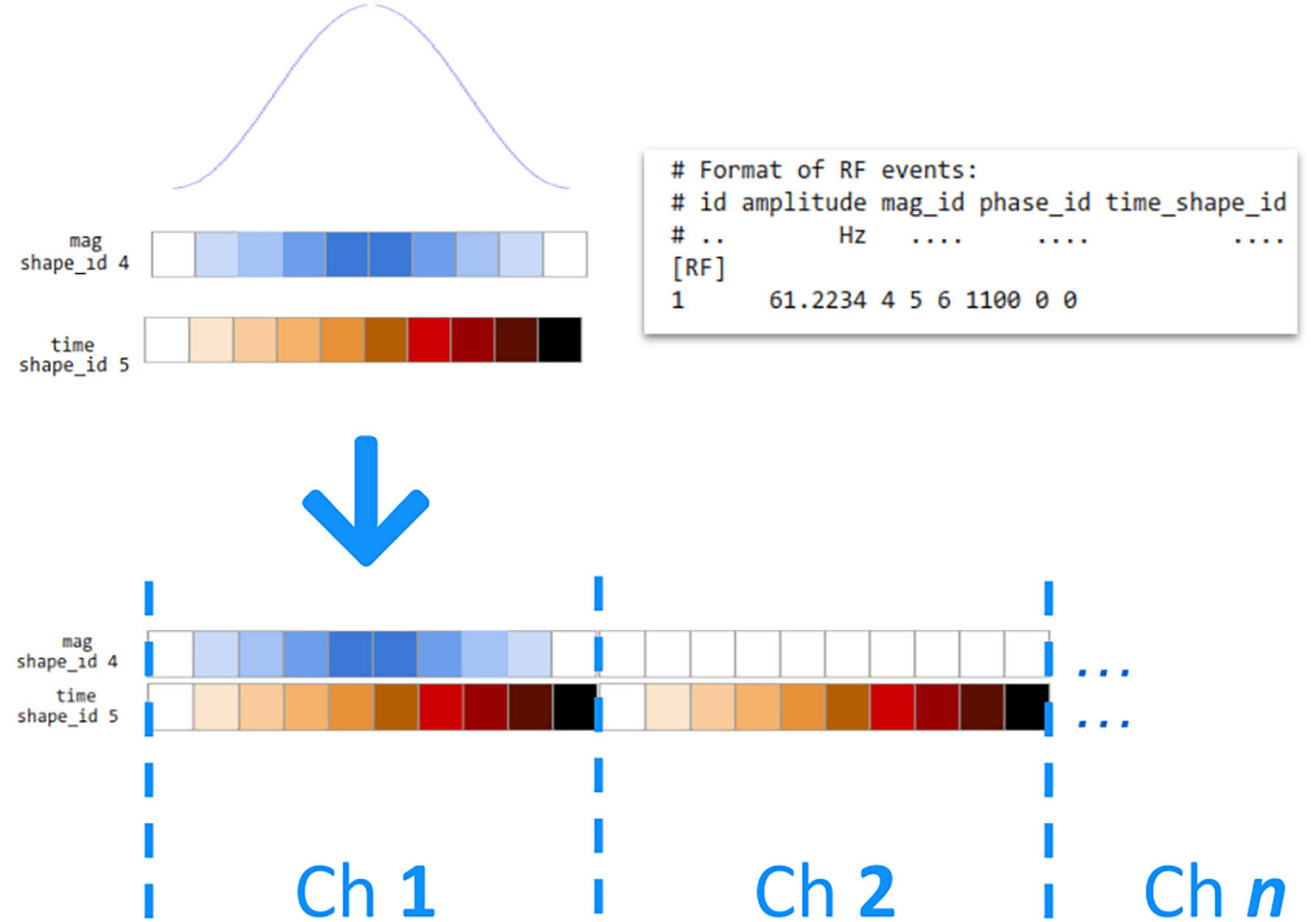
Schematic depiction of how a conventional non‐parallel‐transmit (pTx) radiofrequency pulse (*top*), featuring a Sinc magnitude shape and linear time shape, is transformed into a pTx pulse (*bottom*) by concatenating all channels and repeating the time points. In this example, only the first transmit channel is used, corresponding to the left‐most slices depicted in Figure 6.

The Pulseq specification supports RF events with waveforms for magnitude, phase, and time.^15^ The latter is intended to efficiently store pulses by only storing time points with changes, but it can also be used to store repeated time points. We leverage this by repeating the time range for each transmit channel , enabling the magnitude and phase waveforms to contain all channels while still allowing for effective waveform compression. All channels must end with the latest time point used in any one channel, as both the current reference C++ library (v1.5.0) and our interpreter use the final time point to decompress the waveforms and determine their duration. A visualization of how the contents of each channel is repeated within the shape is available in Figure 1.

#### 2.1.2 $B_{0}$ orientation conversion

Converting pTx pulses for compatibility between different MRI platforms requires addressing potential variations in hardware and system conventions, such as RF channel ordering, $B_{0}$ field direction, and gradient axis system conventions. In Pulseq, gradient channels are directly mapped to the hardware, and in pTx‐Pulseq, RF channels follow a similar hardware mapping. These mappings can differ between platforms and may require reordering.

The $B_{0}$ direction affects the ${\tilde{B}}_{1}^{\left(+\right)}$ field patterns produced by each RF channel. Although non‐pTx pulses use a circularly polarized combination of channels that yields the same B_0_ regardless of B_0_ orientation, this is not the case for the individual channels used in pTx. However, pTx pulses can be converted when designed for setups that exhibit a symmetry in the ${\tilde{B}}_{1}^{\left(+\right)}$ sensitivities across the transverse plane. In practice, such setups typically include the coil and its interaction with the patient. Minor asymmetries, such as a slight rotation of the patient's head, are expected to result in similar levels of error regardless of the $B_{0}$ direction, making pulse conversion an effective approach for these cases. In setups without such symmetry, instead of converting the pulse, reversing the orientation of the subject inside the magnet (feet‐first instead of head‐first, or vice versa) could be an option, as that results in an effectively reversed $B_{0}$ direction. However, this approach is impractical for applications like brain scans with a fixed head coil setup—as is the case in this work.

For this work, the PASTeUR pulses developed for Siemens 7T systems were adapted to the Philips 7T platform. The Appendix A contains the full description and derivation of this $B_{0}$ direction conversion process and highlights other practical aspects that might need to be accounted for. For example, the axis system conventions for pTx pulses differ between Siemens and Philips scanners, and Appendix A visualizes these to explain how the gradients are mapped for the pTx pulse to function correctly. After all the conversion steps were applied to the PASTeUR pulses, their waveforms were formatted into a pTx‐Pulseq sequence using the *MATLAB* toolbox as previously described. The converted PASTeUR pulses are made available through the research community of the vendor (more information in the Data Availability Statement).

### 2.2 Pulseq interpreter for Philips

#### 2.2.1 Pulseq‐only sequences

In the development platform that Philips offers, the different waveforms and properties of sequence objects, such as gradients and RF pulses, are assigned to a predefined set of objects. These objects expose a subset of their parameters that are optimized for runtime changes, to allow for alterations over repetitions. One approach to support Pulseq would be to detect repetitions and, consequently, statically map Pulseq objects to vendor‐native objects. However, this method requires detectable sequence structures and offers limited sequence flexibility, as larger and/or more complex Pulseq sequences may not be compatible. To achieve the necessary flexibility for a 100% compatible Pulseq interpreter, we adopted a different approach that dynamically maps Pulseq to vendor‐native objects, which are extensively modified during sequence execution.

Within the standard research agreement with our MRI vendor, researchers can collaborate using an online research community and exchange code in a shared repository. In our study, we have created modified versions of the software releases (Versions R5.4 and R5.9). We changed the source code to place one RF object, 300 gradient objects for each axis, one trigger object, and one acquisition object in a spectroscopy free induction decay (FID) sequence. During repeated executions of this sequence, these objects are modified using the reference C++ Pulseq interpreter from GitHub (v1.4.1 for R5.4 and v1.5.0 for R5.9) to recreate the Pulseq sequence step by step. This overwrites the spectroscopy FID sequence, essentially treating it as a “dummy” sequence, and results in a sequence that matches the Pulseq file. The scan parameters that are not overwritten by the Pulseq sequence, such as the coil selection, shimming and dynamics, remain fully functional. The customized files will be shared through a specialized repository for research sites, available at openmr.nl (more information in the Data Availability Statement).

The modified source code has been compiled using the vendor's pulse program environment and copied to the patch folder of the 7T Achieva MRI scanner. A patient is registered in the MRI system, and a default spectroscopy sequence is selected, with the newly introduced “Pulseq” flag set active and a (.seq) file chosen. When this sequence is run, the Pulseq interpreter takes full control of the 7T Achieva MRI scanner.

Because Philips is one of the later platforms to gain Pulseq support, the currently available Pulseq sequences are not designed with the hardware timings of the Philips platform. To make as many of the already available sequences accessible on the platform, the interpreter is optimized for both flexibility and accuracy in timing.

Therefore, the trapezoid and “extended trapezoid” gradients of Pulseq are converted to Philips‐native trapezoid gradients, which do not impose any timing requirements. However, arbitrary gradients are more restrictive, requiring a 6.4‐us time grid compared with the 10 us used by Siemens systems and common to Pulseq sequences written to date, thus requiring up‐sampling similar to the GE interpreter.^16^

For RF pulses, the interpreter attempts to match the hardware timing to match the selected sequence, with a fallback on the native support for interpolation if needed. Apparent diffusion coefficient (ADC) events are only constrained by the system's maximum bandwidth (sample rate).

For both RF and ADC transmit/receive operations, additional time before and after is required for hardware mode switches. Although Pulseq supports these constraints through its DeadTime and RingdownTime parameters, these times are, unlike on other platforms, not fixed and depend on the specific RF and ADC parameters. Conservative fixed timing settings would work but hinder sequence performance, especially in sequences that contain many consecutive ADCs, such as echo‐planar imaging (EPI).^17^ To allow such advanced sequences to run, the interpreter dynamically minimizes hardware switching by combining multiple Pulseq blocks and removing the need for any Dead‐ or RingdownTime entirely.

#### 2.2.2 Hybrid sequences

**FIGURE 2:**
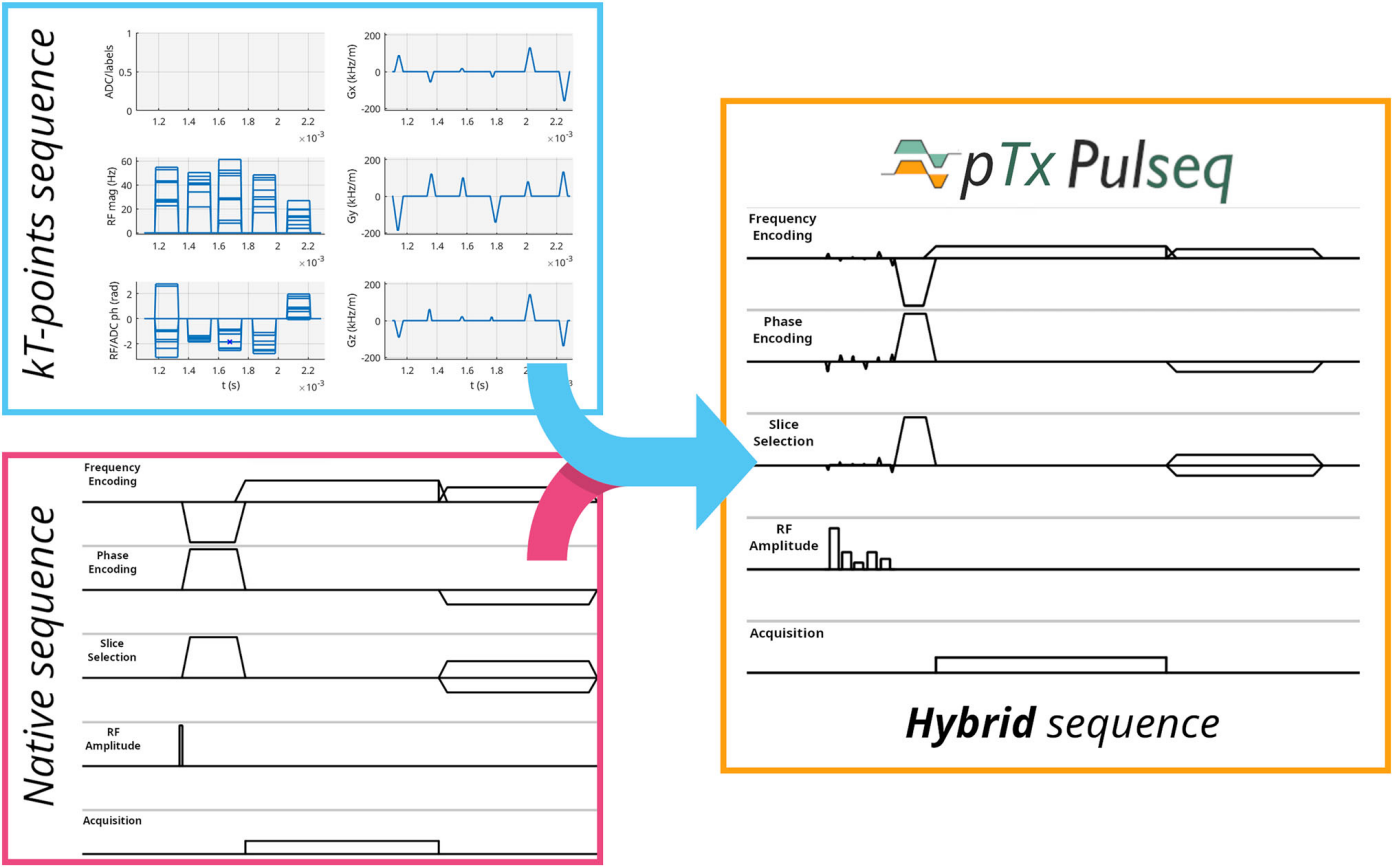
Schematic representation of the hybrid parallel‐transmit (pTx) Pulseq approach, which merges a native vendor‐generated sequence with a Pulseq sequence. In this example, a pTx‐Pulseq sequence containing a kT‐points excitation pulse is merged with a native magnetization‐prepared rapid gradient‐echo sequence by replacing the excitation block pulse with the kT‐points excitation.

In addition to running Pulseq sequences independently, the interpreter also supports hybrid sequences (Figure 2), where Pulseq sequences are integrated with conventional Philips sequences, referred to as native sequences. This hybrid mode is implemented for imaging sequences and replaces or appends native sequence elements with Pulseq sequence blocks. Contrary to the Pulseq‐only mode, the native sequence is not treated as a dummy sequence and is not modified during runtime, but it merged with the Pulseq sequence.

Previous work demonstrated an approach that interleaved Pulseq blocks, with the vendor's native readout module.^7^ Although this technique could potentially offer more flexibility, as the interleaved Pulseq blocks can be completely arbitrary, our merging strategy fully harnesses the flexibility and capabilities offered by the native sequence. As the native sequence structure is retained, only specific sequence elements can be replaced and enhanced. To demonstrate this, we have configured the hybrid mode to remove the vendor's excitation pulse and replace it with a pTx‐Pulseq sequence containing an excitation pulse.

### 2.3 Simulation experiments

To validate the capabilities of our Pulseq interpreter, we conducted a series of simulation experiments using three example sequences from the Pulseq GitHub repository.^18^, ^19^, ^20^ These include the gradient echo (GRE), EPI with ramp sampling, and spiral sequences. Each sequence was chosen to challenge different aspects of the interpreter's capabilities, from basic sequence fidelity in the GRE sequence to the complex timing requirements of the EPI sequence and the handling of arbitrary gradients in the spiral sequence. The simulations are performed in the vendor's pulse programming environment and visualized using the graphical pulse sequence viewer of the environment and compared against the intended waveforms plotted directly by Pulseq for any discrepancies.

### 2.4 Safety validation

The scanner's real‐time safety systems have not been altered in the patched software. This includes the power monitoring for specific absorption rate (SAR) limits and system performance, as well as safety limits such as gradient specifications (max strength, slew rate) and temperature monitoring. These essential safety systems are validated for their correct operation with the Pulseq interpreter by attempting to run a sequence that exceeds the allowed system specifications and SAR limits and asserting the scanner safely aborts.

Because the interpreter modifies the dummy sequence during runtime, some of the vendor's validation steps need to be corrected, as they are performed on the dummy sequence and not the actual Pulseq sequence. This includes the SAR prediction, the gradient resonance and sound pressure level prediction, and the peripheral nerve stimulation estimation. Although we aim to restore the native on‐scanner functionality in future versions of the interpreter, we can run these algorithms in *MATLAB* leveraging the Pulseq library. The *MATLAB* scripts require scanner‐specific hardware files and read a selected Pulseq sequence file to perform said predictions. The scripts then write both the results, in addition to the scanner‐specific parameters used in the calculation, in the definitions header field of the sequence file. This allows the interpreter to finalize the safety predictions if the scanner‐specific parameters match and display the correct prediction results. Additionally, as the Pulseq interpreter from v1.5.0 onward supports the verification of the sequence file through its cryptographic hash, these safety predications cannot be unintentionally corrupted.

The *MATLAB* scripts that read the scanner‐specific hardware files and perform the algorithms will be shared as part of the interpreter (more information in the Data Availability Statement).

### 2.5 Field measurements

Recognizing the potential inaccuracy of the earlier simulation‐based validations in the vendor's pulse programming environment, especially given the runtime manipulations performed by our Pulseq interpreter, we conducted field measurements to verify the accuracy of the actual gradient waveforms produced.^21^ These measurements were carried out by placing a 16‐channel field camera (Dynamic Field Camera, Skope, Switzerland) in the center of the bore and running the three types of previously simulated example sequences from the Pulseq GitHub. The measured k‐space trajectories and gradient waveforms are plotted and checked for consistency with the requested trajectories and waveforms directly from the Pulseq files.

### 2.6 Imaging experiments

Imaging experiments were conducted on a 7T Achieva MRI scanner (Philips, The Netherlands), running either a version of the R5.4 or the R5.9 release that is patched to include the Pulseq interpreter with support for pTx‐Pulseq and hybrid sequences. All experiments used an 8‐channel transmit/receive and 32‐channel receive head coil (Nova Medical, USA), effectively creating a 40‐channel receive system.^21^, ^22^

#### 2.6.1 Phantom scans

For the phantom scans, we used either a 100‐mm spherical phantom or a head‐shaped phantom. Each was centered within the RF coil and positioned at the magnet's isocenter. We acquired a two‐dimensional GRE image with a field of view (FOV) of 256 × 256 mm^2^ at 1‐mm resolution and 3‐mm slice thickness and echo time (TE)/repetition time (TR)/α/bandwidth = 6 ms/15 ms/6°/900 Hz/px. The scan performed on the head‐shaped phantom incorporated pTx, activating each of the eight transmit channels individually across eight repeated slices. Each scan was acquired on R5.4 using both a Pulseq sequence and a parameter‐matched native sequence with native pTx functionality. Images were reconstructed from k‐space data exported from the scanner, read into, and processed using *MATLAB* via Reconframe (Gyrotools, Switzerland). Raw data were sorted using the vendor's acquisition labels for native scans or with labels directly from the Pulseq sequence file. Both scans are reconstructed by a two‐dimensional inverse Fourier transform and root‐sum‐of‐squares coil combination. Additionally, real‐time power monitoring data were captured and visualized using the vendor's viewer from an extended Pulseq sequence that activated two channels simultaneously to visualize pTx control and validate power monitoring.

#### 2.6.2 In vivo scans

In vivo scans were performed on a healthy volunteer who provided informed consent as per local institutional review board guidelines. Two sets of magnetization‐prepared rapid gradient echo (MPRAGE) T_1_‐weighted scans were acquired: one to allow for a direct comparison of sequence fidelity and a second set to harness the capabilities of pTx‐Pulseq in a hybrid sequence.^23^

The first set was acquired using R5.9 and consists of a Pulseq scan that was generated using the accelerated MP‐RAGE sequence example from the Pulseq Github repository, and a parameter‐matched native version.^23^ The Pulseq sequence was adapted for 7 T by setting inversion time/TR_shot_/α/bandwidth to 1.2 s/3.5 s/6°/250 Hz/px, and lengthening the adiabatic inversion RF pulse. Sequence parameters, such as the TE and TR, which are chosen by the Pulseq sequence example, were matched on the native sequence, resulting in a FOV of 256 × 192 × 240 mm^3^ at 1 mm^3^ that is acquired using an acceleration factor of 2× and parameters TE/TR = 3 ms/6.84 ms. Both the native and the Pulseq scans were read into *MATLAB* and reconstructed using ESPiRIT and 10 iterations of conjugate‐gradient sensitivity encoding through BART's pics command.^24^, ^25^, ^26^

For the second set of scans, a PASTeUR kT‐points excitation pulse was integrated into a native MPRAGE sequence via our Pulseq interpreter in hybrid mode.^27^ This replaced the vendor's native excitation pulse with the pTx‐Pulseq sequence. The MPRAGE scans were acquired using both the vendor‐provided block pulse and the pTx‐Pulseq PASTeUR excitation module. Although this set of scans had an FOV identical to that of the first set, these were accelerated 4 times using compressed sensing and used parameters TE/TR = 3.3 ms/9 ms. The second set of scans were acquired using R5.4 and reconstructed online using the vendor's reconstruction methods.

## 3 RESULTS

The source code files, adapted to include our interpreter, were successfully compiled without any warnings and subsequently loaded as a “patch” onto both the simulator and MRI system using the research account. The MRI system started with the only warning that a patched system was observed and that the MRI vendor cannot be held responsible for unintended use. After acknowledging and accepting this warning, the “Pulseq enable” switch and sequence file (.seq) selection parameters are available, indicating integration of our interpreter into the MRI system.

The correct operation of all real‐time safety systems was verified; the Pulseq sequence that intentionally exceeds the system specifications was aborted, and the Pulseq sequence that uses too many large flip‐angle pulses similarly halted and raised the power monitoring dialogue. The real‐time interlock of the gradient system was simulated by overriding a gradient coil temperature sensor during a Pulseq scan, which also resulted in the scanner correctly aborting.

### 3.1 Simulation experiments

**FIGURE 3:**
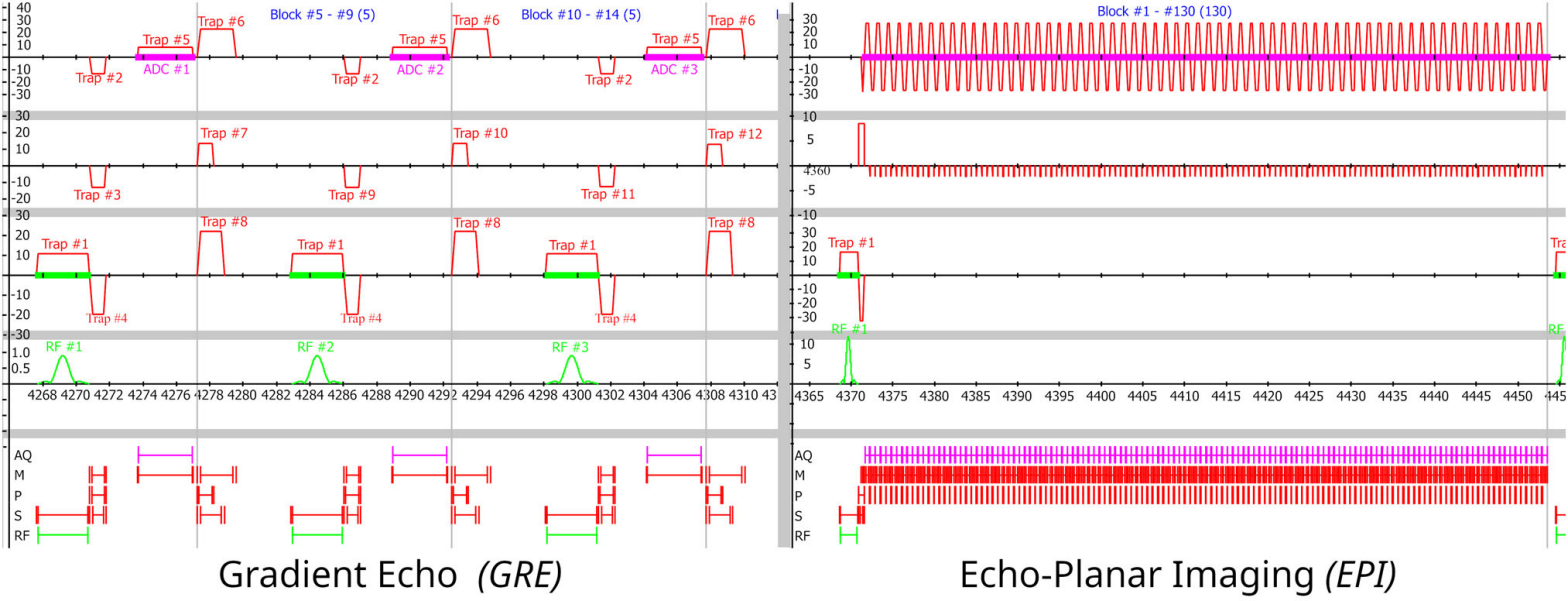
Pulse sequence diagrams of a gradient‐echo sequence (GRE; *left*) and an echo‐planar imaging (EPI; *right*) sequence, resulting from spectrometer simulations while running the Pulseq interpreter. Both sequences are based on the example sequence files from the Pulseq GitHub repository^18^, ^19^ and are visualized using the vendor‐provided graphical viewer. The specific EPI sequence simulated uses ramp sampling, which demonstrates the interpreter's support for short dead time between consecutive readouts. The Pulseq interpreter can dynamically update the text labels of objects, renaming them according to the Pulseq events.

All three Pulseq sequences were executed successfully in the simulation environment. The simulations of the MRI spectrometer resulted in vendor‐specific logs that have been visualized through the use of the accompanying vendor‐provided graphical waveform viewer. Figure 3 shows the simulated GRE and EPI sequences matching exactly to the requested sequence and waveforms as defined in the Pulseq sequence file.^18^, ^19^ The spiral sequence,^19^ and specifically the non‐trapezoidal gradient waveforms it contains, are runtime changes to the internal vendor‐native objects that are normally not supported by the vendor's simulation tooling. Initially, these gradient waveforms did not show correctly in the vendor‐provided simulation and visualization tools. However, after extending the simulator to support runtime changes, this functionality was restored as shown in Figure S1.

### 3.2 Field camera measurements

**FIGURE 4:**
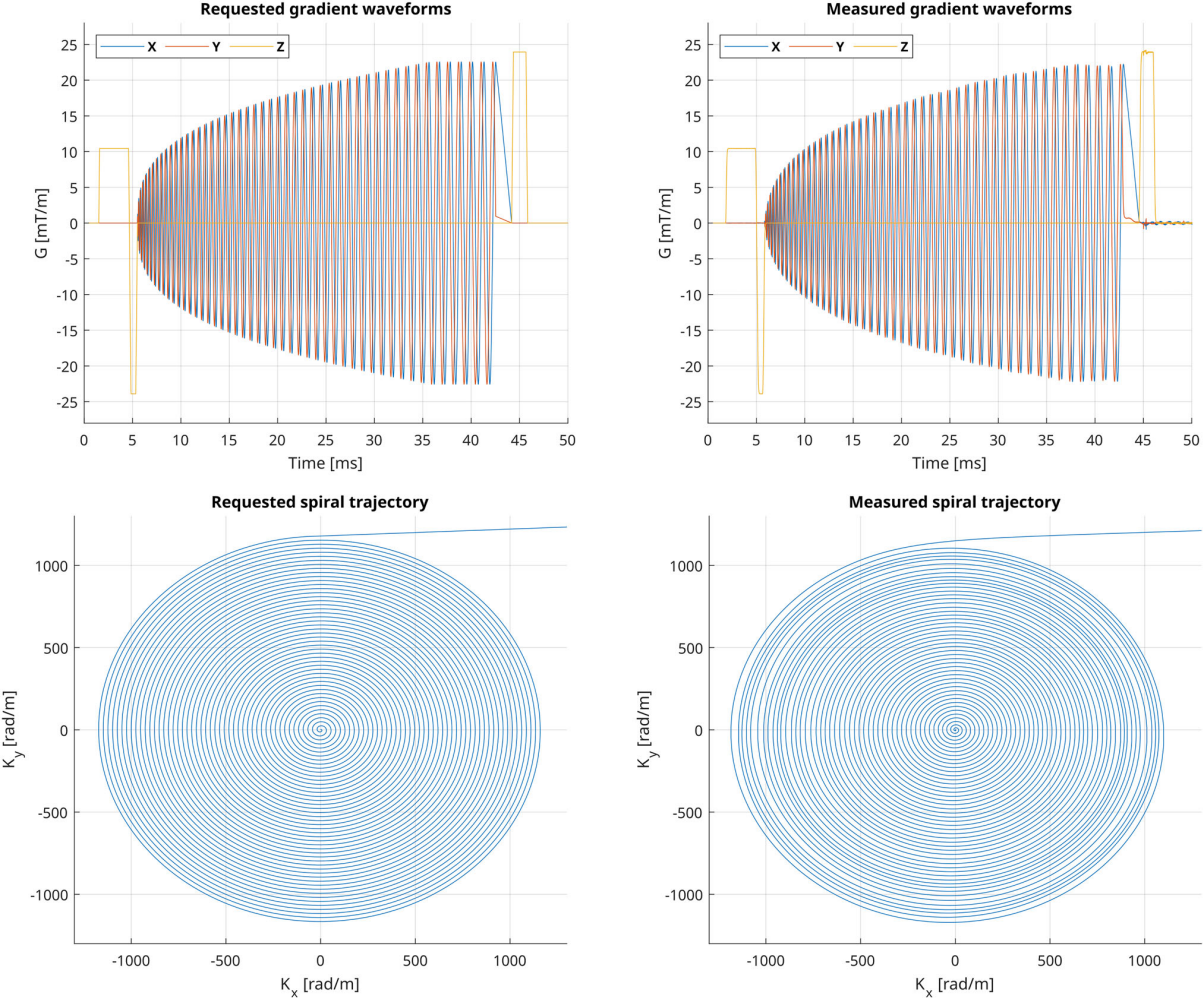
Gradient waveforms (*top*) and k‐space encoding trajectory (*bottom*) of a spiral readout, shown both as requested in the Pulseq sequence definition (*left*) and as captured by the field camera (*right*). This spiral trajectory is part of the spiral example sequence from the Pulseq GitHub repository.^20^

To validate the actual gradient waveforms produced by the sequences, the sequences were run on the MRI scanner and measured with the field camera. All trajectories were measured up to the level of the final gradient crusher, which caused spins in the field probes to dephase to the level that SNR was insufficient to assess the field.^21^ The field camera could not only characterize the GRE and EPI sequences, but also the correct spiral trajectory, as shown in Figure 4. The requested gradients in the spiral Pulseq sequence file are shown next to the gradients captured by the field camera in Figure 4 and show only small discrepancies that are expected with a spiral sequence.^28^, ^29^

### 3.3 Imaging experiments

Figure S2 provides an animation of the monitoring system running the extended pTx‐Pulseq sequence. This animation highlights the dynamic pTx control that pTx‐Pulseq enables.

#### 3.3.1 Phantom scans

**FIGURE 5:**
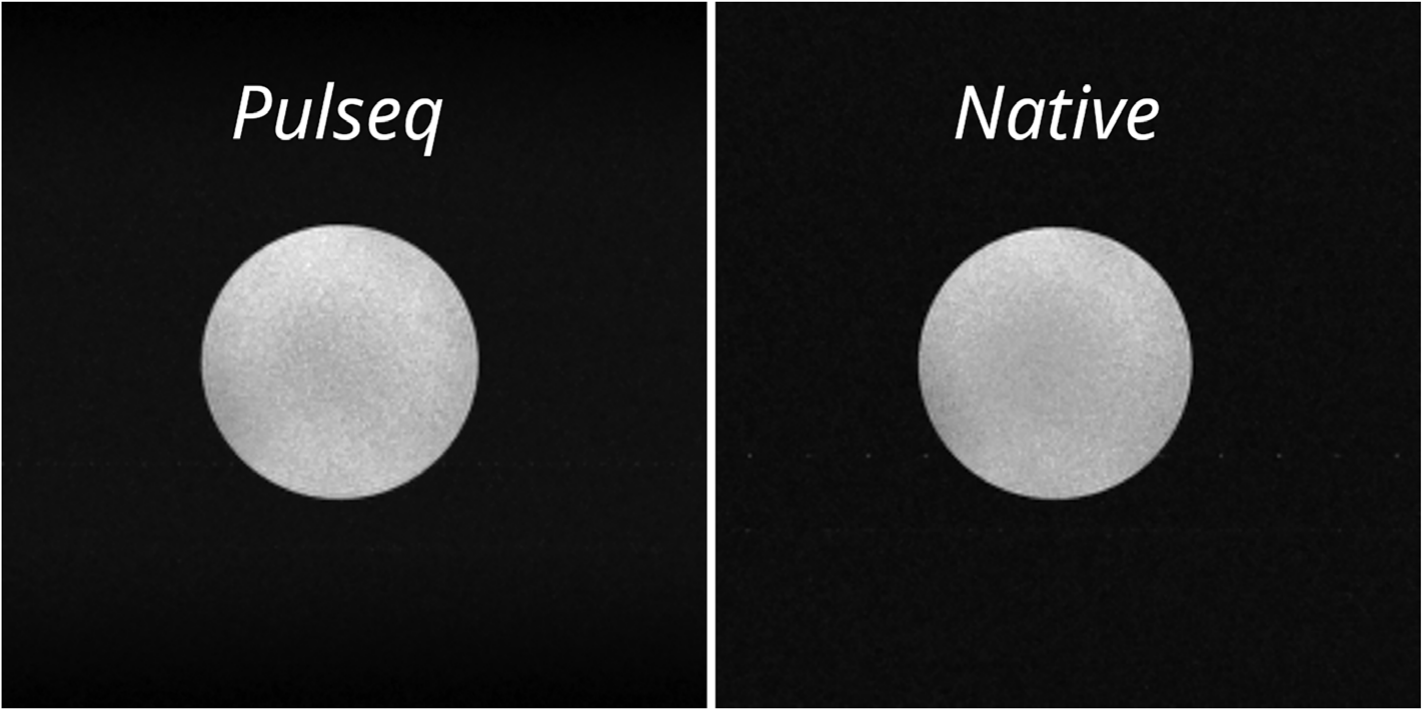
Reconstructed images from the Pulseq gradient‐echo sequence (*left*) and a native gradient‐echo sequence (*right*), with sequence parameters matched to the Pulseq implementation. The gradient‐echo Pulseq sequence was adapted from an example sequence available on the Pulseq GitHub repository.^18^

Figure 5 shows the reconstructed slices from the non‐pTx GRE scan performed on the spherical phantom. This scan shows minor inhomogeneities in both the Pulseq sequence and the parameter‐matched native sequence, which are attributable to the inhomogeneous B_1_^+^ field at 7 T and the receive sensitivity profile of the head coil.^9^

**FIGURE 6:**
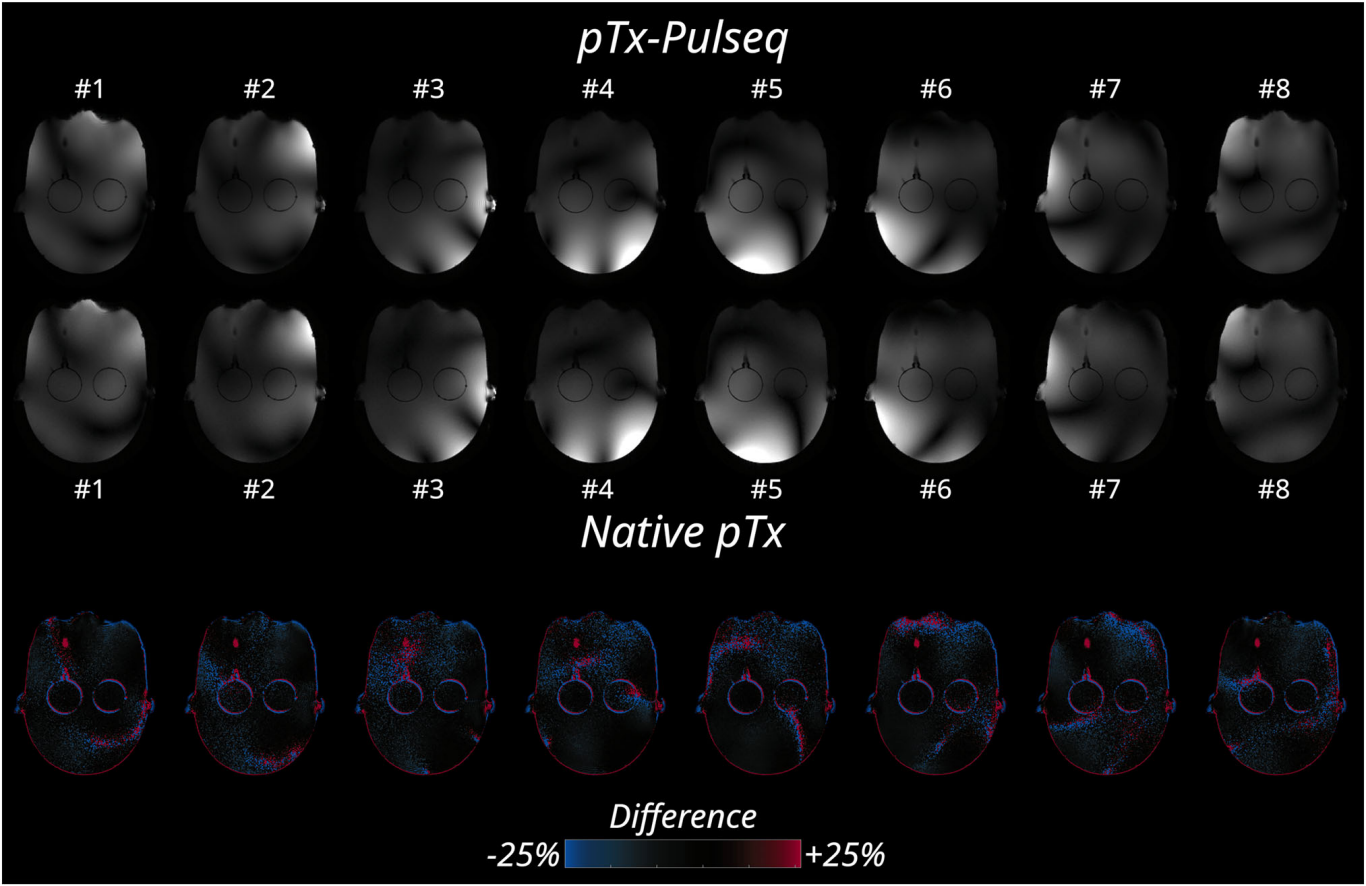
Single transmit‐channel scans acquired using parallel‐transmit (pTx) Pulseq (*top row*) and the vendor's native pTx (*middle row*) are compared, with the difference maps (*bottom row*) highlighting the relative change, shown as a percentage. Each of the eight transmit channels show a high degree of similarity between the two implementations. Both sets of scans display nearly identical ${\tilde{B}}_{1}^{\left(+\right)}$ spatial patterns, highlighting the correct control over all transmit channels individually through pTx‐Pulseq. The small differences visible near geometrical boundaries and in regions with low signal‐to‐noise ratio (e.g., areas with low ${\tilde{B}}_{1}^{\left(+\right)}$) are expected, given the slight differences in the excitation profiles resulting from differing pulse shapes.

The pTx‐enabled GRE scan performed on the head‐shaped phantom is depicted in Figure 6. In this Figure, the spatially identical ${\tilde{B}}_{1}^{\left(+\right)}$ patterns across all slices indicate correct control over all transmit channels through pTx‐Pulseq. The observed image signal intensity and SNR between the Pulseq and native versions are similar in both Figures.

#### 3.3.2 In vivo scans

**FIGURE 7:**
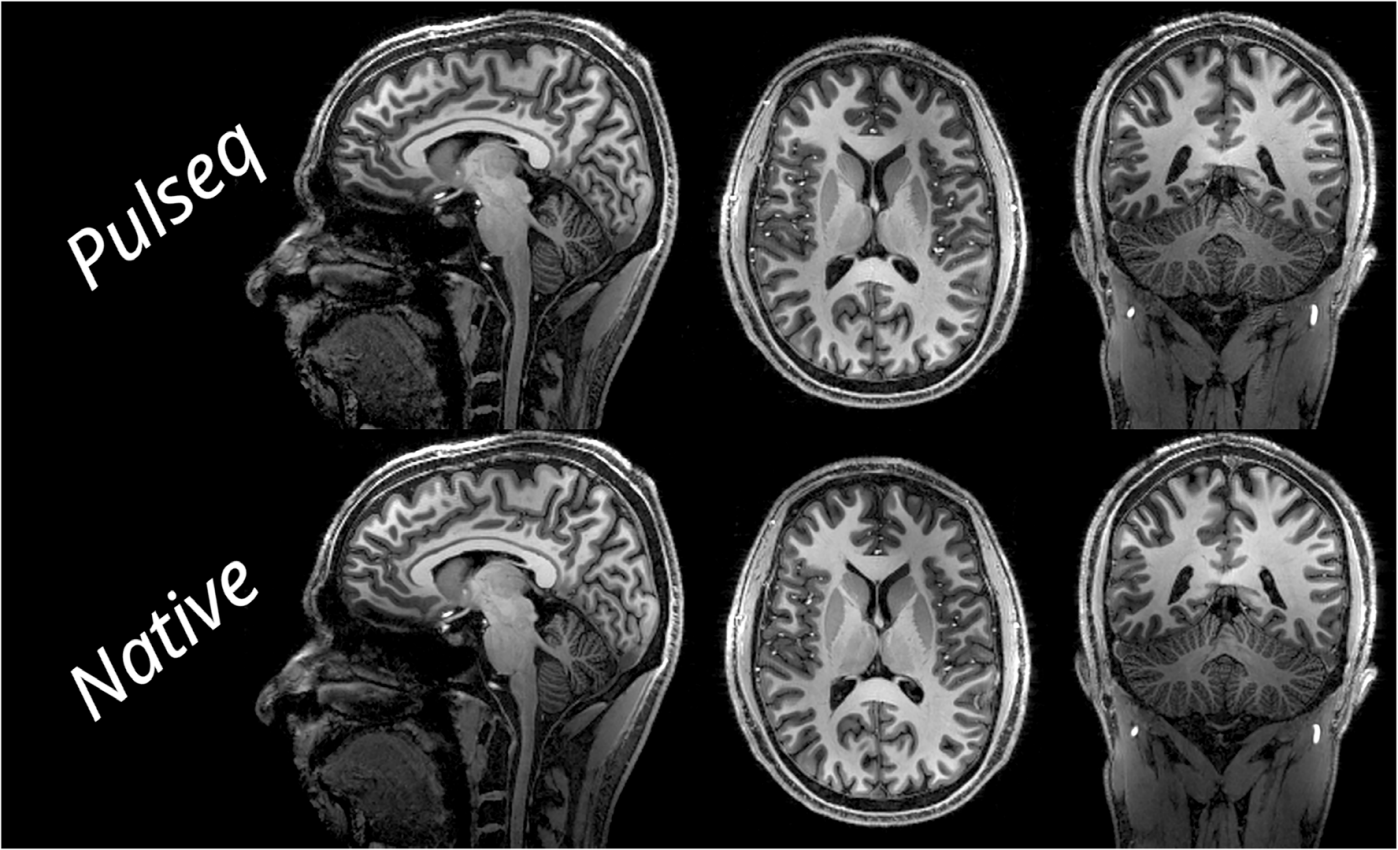
Three slices of a magnetization‐prepared rapid gradient‐echo scan acquired as either a Pulseq sequence (*top*)^23^ or as a parameter‐matched native sequence (*bottom*). Both scans are reconstructed offline using coil sensitivity maps computed using ESPiRIT and through BART's conjugate‐gradient sensitivity encoding implementation.^24^, ^25^, ^26^ The close match in contrast and signal‐to‐noise ratio suggest that the Pulseq sequence closely matches the native implementation.

The first set of MPRAGE scans depicted in Figure 7, which are acquired using either a Pulseq sequence or a native sequence, are virtually indistinguishable from each other. Although some areas show minor differences due to subject motion, both the contrast and SNR are very similar.

**FIGURE 8:**
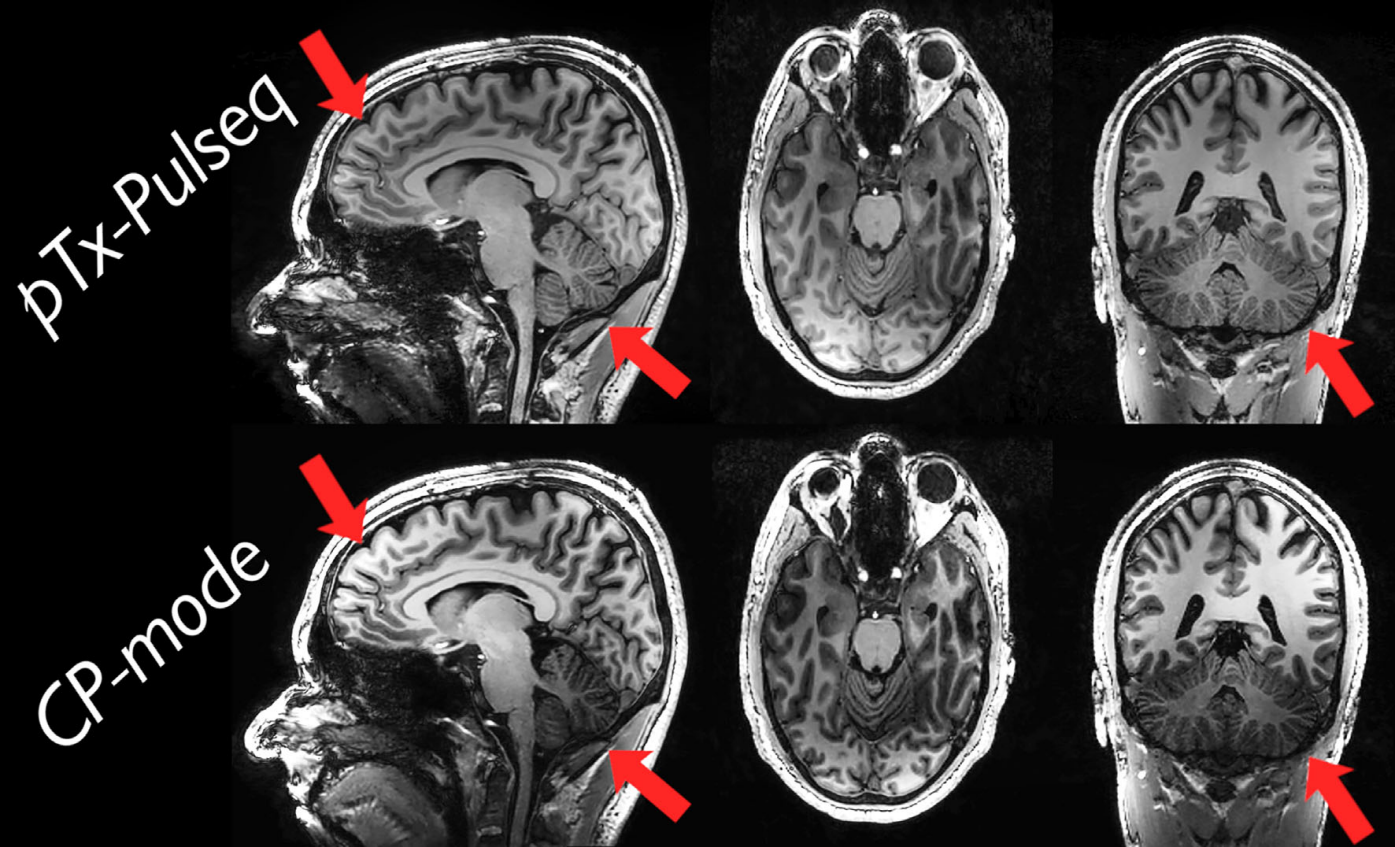
Three slices of a native magnetization‐prepared rapid gradient‐echo sequence acquired with either a parallel‐transmit (pTx) Pulseq universal pulse (UP) excitation from the PASTeUR package (*top*)^36^ or with a regular nonselective excitation circular polarized (CP) mode (*bottom*). The hybrid approach enhances the vendor's optimized sequence by merging the pTx‐Pulseq sequence, which contains the UP excitation, into the native sequence by replacing its excitation pulse. Both scans are reconstructed by the scanner using the same compressed sensitivity encoding and inhomogeneity correction techniques. The red arrows highlight regions with improved excitation uniformity in the pTx‐Pulseq images: Anterior cortical areas that appeared overly bright in CP mode are more balanced, and the cerebellum, previously affected by low ${\tilde{B}}_{1}^{\left(+\right)}$, shows improved signal homogeneity.

In Figure 8, the effect of replacing the nonselective excitation of a native MPRAGE scan with the PASTeUR pulse using our Pulseq interpreter in hybrid mode is shown. The pTx pulse improved the overall homogeneity of the image while not negatively affecting the rest of the scan. Especially in the areas that suffer from a low ${\tilde{B}}_{1}^{\left(+\right)}$ with the native excitation, such as the cerebellum, the pTx pulse improved signal‐to‐noise and contrast‐to‐noise ratios.

## 4 DISCUSSION

In this work, we have extended the Pulseq ecosystem to Philips MRI scanners by developing the first Pulseq interpreter for this platform and by introducing two extensions to the framework itself. First, pTx‐Pulseq brings full dynamic pTx capabilities to Pulseq without requiring changes to the existing format. Second, hybrid sequences enable the integration of Pulseq with vendor‐optimized sequence components. We validated the practicality and effectiveness of these innovations through field measurements, safety assessments, and both phantom and in vivo imaging at 7 T. To showcase their potential, we converted a UP from the PASTeUR package—originally made for Siemens systems^21^, ^24^—into the pTx‐Pulseq format, and ran it on a Philips 7T MRI scanner within a hybrid sequence. We believe this represents the first successful conversion and cross‐vendor application of UPs.

Recent studies have shown that it is possible to run Pulseq sequences by converting them to a format closer to the native structure.^30^ Although that might seem easier initially, our runtime approach provides optimal compatibility and flexibility without requiring pre‐conversion. Our interpreter not only highlights the Philips hardware's capability for substantial runtime flexibility but also the vendor‐native platform's adaptability, as it can accommodate most of the timing parameters used in existing Pulseq sequences.

Pulseq is one of several open‐source frameworks available for MRI sequence development.^2^, ^3^, ^4^, ^5^ Although our implementation was tuned for Pulseq, an almost identical approach is envisioned for merging the other frameworks into the vendor‐native platform.

The need for pTx capabilities in Pulseq has driven earlier efforts to add support for static pTx.^14^ These efforts involved adding new entry fields to the sequence format—a modification incompatible with the current Pulseq specification that would necessitate a revision of the specification. Current implementations of the interpreter might fail in a fail‐safe manner and reject the sequence, or they could potentially misinterpret the sequences. In contrast, our development does not alter the fundamental Pulseq file format, thus maintaining compliance with the current specification. Moreover, efforts are already being made to implement this pTx‐Pulseq approach in systems from Siemens, highlighting the adaptable nature of our solution.

Reconstructed images from Pulseq sequences on a Philips MRI scanner demonstrated comparable image quality to those obtained from native sequences, confirming the successful integration of our Pulseq interpreter. Although this initial implementation and demonstration was performed on a 7T system, it is not restricted to 7T systems and can be compiled for clinical 1.5T and 3T MRI scanners.

The hybrid mode allows researchers to exploit the full capabilities of Pulseq for specific sequence modules, such as a prepulse or a different excitation while maintaining the use of all the vendor's optimized encoding and image‐reconstruction functionality. For example, ${\tilde{B}}_{1}^{\left(+\right)}$ “insensitive” prepulses, such as TR‐FOCI (inversion),^26^ can be directly compared or swapped into existing protocols to evaluate their performance. Similarly, sequences can be enhanced through using dead‐time periods, such as incorporating fat navigators to add motion correction.^31^ This flexibility allows researchers to avoid the complexity of recreating entire sequences, thereby making advanced pulse‐sequence design through Pulseq more accessible.

In the hybrid mode, our Pulseq interpreter statically translates Pulseq sequences into the native sequence format rather than dynamically modifying a native dummy sequence. As a result, it ensures that all standard safety checks and simulations are conducted accurately. It also lacks the flexibility provided by the Pulseq‐only mode. Moreover, using the hybrid mode effectively requires a certain level of familiarity with the native sequence architecture, as correct placement of Pulseq modules within these sequences is needed for their use. Although the interleaved approach of previous work could potentially allow a greater level of per‐TR flexibility,^7^ our merging strategy is able to harness the native sequence structure and retain the flexibility to change its parameters. Additionally, it offers the possibility to enhance all components of the sequence, including the readout itself, which might not be possible in the interleaved approach.

Advanced RF pulse techniques have demonstrated significant benefits in 7T MRI systems and are likely to become essential in higher‐field scanners, according to recent studies.^32^ The Pulseq framework, enhanced by our hybrid mode and pTx capabilities, enables the effective dissemination of pTx pulses across various sequences and MRI systems, paving the way for their standardization. The benefits of this approach are not only applicable for pTx pulses but apply to all components of modular sequence designs. The Pulseq‐CEST project, which only provides CEST‐preparation blocks, demonstrates this effectively. By defining standardized positions in sequences and standardized interleaving paradigms, interpreters can add support for such focused projects. Future initiatives could formalize the hybrid use of Pulseq sequence into the Pulseq specification itself. Such standardization could streamline research processes and facilitate the widespread adoption of advanced techniques, ensuring consistency and comparability of results across different systems and studies.

With the introduction of pTx‐Pulseq, functionality previously confined to vendor‐specific environments is now readily accessible, highlighting Pulseq's flexibility and extensibility.

Future developments could further broaden Pulseq's capabilities, such as by enabling control over more than three gradient axes to facilitate the use of insert gradients, which are specially designed gradients for targeted applications such as brain imaging. Previous research with insert gradients used an external waveform generator to generate a single repeated waveform during a sequence, which limited the application to static parts of the sequence.^33^ Incorporating the ability to use Pulseq for sequences with more than three gradient channels would allow playing out dynamic waveforms on insert gradients. This would allow researchers to integrate these specialized gradients seamlessly into any part of their MRI sequences, further expanding the clinical and research applications of MRI technology.

By making Pulseq compatible with Philips MRI scanners, we can reach out to a significant portion of the global MRI market, enhancing the framework's relevance and applicability. The collaboration of the four largest MRI vendors under Pulseq's umbrella sets the stage for multicenter trials that leverage diverse MRI systems, enabling the standardization and dissemination of novel MRI sequences—or parts of sequences through the hybrid paradigm.

The next logical step in leveraging this widespread adoption is to establish a regulatory framework that supports the clinical approval of open MRI sequences. Such a framework would enable the use of Pulseq‐enhanced MRI systems in clinical trials, potentially accelerating the translation of advanced imaging techniques from research to routine clinical practice.

## 5 CONCLUSION

This study has successfully extended the Pulseq framework to Philips MRI systems, incorporating dynamic pTx capabilities and hybrid sequences without altering the existing Pulseq format. This development demonstrates the feasibility of cross‐vendor UP applications and highlights the potential for Pulseq to standardize MRI sequence development across different platforms. The collaboration among major MRI vendors under the Pulseq framework not only facilitates the reproducibility and accessibility of advanced MRI techniques for researchers but also sets the stage for rapid clinical translation of innovative imaging techniques, ultimately benefiting both the research community and patient care.

## Supporting information


**Figure S1.** Pulse sequence diagrams of a spiral sequence, resulting from spectrometer simulations while running the Pulseq interpreter. The sequence, sourced from the Pulseq GitHub repository,^20^ is visualized using the vendor‐provided graphical viewer. Gradient waveforms are depicted in two coordinate systems: MPS (*top*) and XYZ (*middle*).

**Figure S2.** Animation of real‐time average radiofrequency (RF) power monitoring during a parallel‐transmit pTx‐Pulseq scan, highlighting the dynamic control over the different transmit channels. The pTx‐Pulseq sequence ran during this animation consists of 20 two‐dimensional (2D) gradient‐echo (GRE) slices acquired with either one or two transmit channels active: first in increasing channel number, then decreasing, and finally the combination of first two sets. The active transmit channel is represented by an increase in the time‐averaged power, as shown in the power monitoring. This level will subsequently decrease when the channel is no longer active.

## Figures and Tables

**FIGURE A1:**
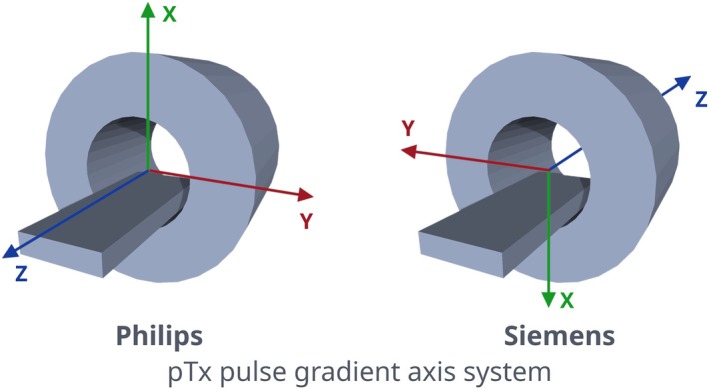
Schematic illustration of the parallel‐transmit (pTx) pulse gradient axis systems for Philips (*left*) and Siemens (*right*) MRI scanners. The Philips system defines the x‐axis as down→up (*green*), y‐axis as left→right (*red*), and z‐axis as back→front (*blue*), from the perspective as viewed when standing in front of the scanner. In contrast, while the Siemens system maintains the same axes, it uses the opposite directional convention. The XYZ terminology in this figure refers to pTx pulses defined independently of the FOV and subject orientation, and may not match the vendor's convention.

## Data Availability

The modified Philips source code that integrates the Pulseq interpreter, together with the supporting *MATLAB* code, will be made available through the vendor's platform linked at openmr.nl. The pTx‐Pulseq sequences used in this work are available through GitHub on https://github.com/Roosted7/ptx‐pulseq. Specifically, commit da510e1 contains both the *MATLAB* script that generates the pTx‐Pulseq sequences as well as the (.seq) files themselves. In addition to the conversion into the Pulseq format, the PASTeUR pulses were also converted into Philips' native external pulse format (.pd and .gd). These versions can be used on any of the pTx‐capable hardware and software releases, without the need for a patch. These pulses, along with “PulseTool” for easy pulse selection and matching with protocols, have been made available on the vendor's research forum for research sites, linked at openmr.nl. The data that support the findings of this study are available from the corresponding author upon reasonable request.

## APPENDIX A PTX PULSE CONVERSION

This Appendix provides a rigorous explanation of why, when converting parallel‐transmit (pTx) pulses between MRI systems with opposite $B_{0}$ field directions, it is necessary to:

1. Reorder radiofrequency (RF) channels over a symmetry axis;
2. Complex‐conjugate the RF waveforms; and
3. Invert gradient waveforms on the nonsymmetry axes.

Before going over these three steps, we will briefly go over other steps that might be needed and the symmetry precondition. This work uses the notation proposed by Hoult.^32^ Quantities in the rotating frame are denoted with a tilde (˜), and this frame can be positive or negative, indicated by superscripts ^(+)^ or ^(−)^. When a formula holds for both the positive and negative frame, the ^(±)^ symbol is used instead of providing both versions separately. Complex quantities are denoted in a cursive font (e.g., ${\tilde{B}}_{1}^{\left(+\right)}$).

pTx pulses can be designed “universally,” such that they will perform well for a certain coil and setup and do not require the prescans and pulse‐design process that is required for per‐subject tailored pTx pulses. These universal pulses have been shown to function well, even for subjects or scanners it was not designed on.^11^

### A.1. Required conversion steps

When playing out a pTx pulse on a different MRI platform (e.g., version, model, brand) than the pulse was designed for, it is likely that some kind of conversion is necessary for the pulse to function as intended. This conversion process may include straight‐forward conversion steps, such as:

- Converting to a different file format;
- Resampling the waveforms to a different dwell time;
- Adding or removing CP‐mode phase to the RF phase;
- Remodulating the RF phase to compensate for differences in table height;
- Reordering the RF and gradient channels; and/or
- Scaling and/or converting the unit of the RF and gradient waveforms.

When the pTx pulse is designed for an MRI system in which the $B_{0}$ field is oriented in the opposite direction, such steps might be needed but are not sufficient to give the intended excitation. This is because the RF magnetic field ${\tilde{B}}_{1}^{\left(+\right)}$ produced by the same coil is different when the $B_{0}$ direction reverses. In this section, we will derive the conversion process that can be used to convert pTx pulses to the reversed $B_{0}$ direction.

### A.2. Effect of $B_{0}$ field direction reversal

The RF magnetic field ${\tilde{B}}_{1}^{\left(+\right)}$ produced by a pTx pulse is determined by the combined contributions of the N transmit channels and their RF waveforms and coil sensitivity, as follows:

(A1)
$${\tilde{B}}_{1}^{\left(+\right)}\left(r,t\right)=\sum_{n=1}^{N}U_{n}\left(t\right){\tilde{S}}_{n}^{\left(+\right)}\left(r\right)$$

where $U_{n}\left(t\right)$ is the complex RF waveform applied to coil n; ${\tilde{S}}_{n}^{\left(+\right)}\left(r\right)$ is the coil sensitivity of channel n in the positive ^(+)^ frame; and r=[x,y,z] is the spatial position vector.

According to the right‐hand rule, when the thumb aligns with the direction of the $B_{0}$ field, the fingers of the right‐hand curl in the direction of the spin's precession and therefore also the direction of the ${\tilde{B}}_{1}^{\left(+\right)}$ field's rotation. From the external laboratory frame perspective, reversing the direction of $B_{0}$ thus reverses both the precession and the ${\tilde{B}}_{1}^{\left(+\right)}$ field's rotation. When reversing the direction of $B_{0}$, what we define as the positive and negative rotating frames are swapped, altering ${\tilde{B}}_{1}^{\left(+\right)}\to {\tilde{B}}_{1}^{\left(-\right)}$ (and vice versa). Following Hoult, ${\tilde{B}}_{1}^{\left(+\right)}$ can be expressed in terms of its Cartesian and complex lab frame components, as follows:

(A2)
$${\tilde{B}}_{1}^{\left(+\right)}={\tilde{B}}_{1x}^{\left(+\right)}+i{\tilde{B}}_{1y}^{\left(+\right)}=\frac{B_{1x}+iB_{1y}}{2}$$

Similarly, in the negative rotating frame, the RF field is represented by ${\tilde{B}}_{1}^{\left(-\right)}$, as follows:

(A3)
$${\tilde{B}}_{1}^{\left(-\right)}={\tilde{B}}_{1x}^{\left(-\right)}+i{\tilde{B}}_{1y}^{\left(-\right)}=\frac{{\left[B_{1x}-iB_{1y}\right]}^{*}}{2}$$

and ${\tilde{S}}_{n}^{\left(\pm \right)}\left(r\right)$ captures the spatial sensitivity of coil n similarly to the RF field representations of Equations ([A2) and (A3) as follows:

(A4)
$${\tilde{S}}_{n}^{\left(\pm \right)}\left(r\right)={\tilde{B}}_{1x,n}^{\left(\pm \right)}\left(r\right)+i{\tilde{B}}_{1y,n}^{\left(\pm \right)}\left(r\right)$$

Because the coil sensitivities differ between the positive rotating frame ${\tilde{S}}_{n}^{\left(+\right)}\left(r\right)$ and the negative rotating frame ${\tilde{S}}_{n}^{\left(-\right)}\left(r\right)$, the RF field ${\tilde{B}}_{1}^{\left(-\right)}\left(r,t\right)$ produced by the unmodified pTx pulse in the reversed $B_{0}$ direction differs and is not as intended.

### A.3. Symmetry in the setup

Although the coil sensitivities ${\tilde{S}}_{n}^{\left(\pm \right)}\left(r\right)$ are intrinsic properties of the MRI system itself and not something we can change, we are able to modify the RF waveform $U_{n}\left(t\right)$. To address the mismatch in coil sensitivities, we exploit a symmetry in the coil array and setup. Specifically, for our head coil, we have the following symmetrical properties:

- A coil array with N channels that are arranged symmetrically along the left–right (y) axis;
- For each coil n at position y, there is a mirror coil $n^{\prime }$ at position −y; and
- The pTx pulse is intended for an object in the coil that is also symmetric along y, so that $r=\left[x,y,z\right]˜r^{\prime }=\left[x,-y,z\right]$.

As Maxwell's equations are invariant under spatial inversion,^33^ the fields produced by the symmetrically arranged coils are related by

(A5)
$$B_{1x,n}\left(r\right)=B_{1x,n^{\prime }}\left(r^{\prime }\right),B_{1y,n}\left(r\right)=-B_{1y,n^{\prime }}\left(r^{\prime }\right)$$

The negative sign of the new y‐component, $-B_{1y,n^{\prime }},$, in Equation ([A5) matches the spatially flipped y‐dimension of $r^{\prime }$. This symmetry allows us to express one rotating frame in terms of the other; by substituting the $B_{1x}$ and $B_{1y}$ fields of Equation ([A5) into Hoult's Equations ([A2) and (A3), only the complex conjugation remains different between the two frames. Therefore, due to this symmetry and its properties, the coil sensitivities satisfy:

(A6)
$${\tilde{S}}_{n}^{\left(+\right)}\left(r\right)={\left[{\tilde{S}}_{n^{\prime }}^{\left(-\right)}\left(r^{\prime }\right)\right]}^{*}$$

Substituting the symmetry (Equation [A6) into A.1 allows us to express the desired ${\tilde{B}}_{1}^{\left(+\right)}$ field in terms of coil symmetries ${\tilde{S}}_{n^{\prime }}^{\left(-\right)}$ of the opposing rotating frame:

(A7)
$${\tilde{B}}_{1}^{\left(+\right)}\left(r,t\right)=\sum_{n=1}^{N}U_{n}\left(t\right){\left[{\tilde{S}}_{n^{\prime }}^{\left(-\right)}\left(r^{\prime }\right)\right]}^{*}$$

### A.4. Reorder channels over symmetry axis

Although Equation ([A7) produces the desired ${\tilde{B}}_{1}^{\left(+\right)}$ field, it does alter the coil sensitivities ${\tilde{S}}_{n^{\prime }}^{\left(-\right)}$, which is not physically possible. However, because this essentially maps the RF waveform $U_{n}\left(t\right)$ to coil $n^{\prime }$, we can also perform the reordering on the RF waveform itself. Similarly, while we cannot invert the −y present in $r^{\prime }$, we can accept a mirrored excitation pattern over the y dimension:

(A8)
$${\tilde{B}}_{1}^{\left(+\right)}\left(r^{\prime },t\right)=\sum_{n=1}^{N}U_{n^{\prime }}\left(t\right){\left[{\tilde{S}}_{n}^{\left(-\right)}\left(r\right)\right]}^{*}$$

### A.5. Complex conjugation of the RF waveforms

Equation ([A8) almost contains the unaltered ${\tilde{S}}_{n}^{\left(-\right)}$ term that our system physically produces, with only the complex conjugation operation remaining. This complex conjugation can be performed on the RF waveform instead, to leave the inverted phase relationship intact, as follows:

(A9)
$${\left[{\tilde{B}}_{1}^{\left(+\right)}\left(r^{\prime },t\right)\right]}^{*}=\sum_{n=1}^{N}{\left[U_{n^{\prime }}\left(t\right)\right]}^{*}{\tilde{S}}_{n}^{\left(-\right)}\left(r\right)$$

### A.6. Invert gradient‐induced phase

To preserve the excitation profile of the pTx pulse under $B_{0}$ reversal, both the RF phase and the gradient‐induced phase must align. Conjugating the RF waveforms inverts the RF phase, which might include the circular polarized (CP) mode phase, thereby converting it to the correct CP‐mode phase ($+45^{\circ },+90^{\circ },\ldots \to -45^{\circ },-90^{\circ },\ldots$, or vice versa).

Any net RF phase offset in the ${\tilde{B}}_{1}^{\left(+\right)}$ field resulting from the local sum of Equation ([A1), $U_{n}\left(t\right){\tilde{S}}_{n}^{\left(+\right)}\left(r\right)$, across the channels will also be inverted. For example, a $+10^{\circ }$ offset on top of the earlier CP mode phase ($+55^{\circ },+100^{\circ },\ldots$) will become a $-10^{\circ }$ offset relative to the converted CP mode phase ($-55^{\circ },-100^{\circ },\ldots$).

Because pTx pulses may harness the spatial interaction between the phase of the ${\tilde{B}}_{1}^{\left(+\right)}$ field and the gradient‐induced phase to achieve their intended excitation profile, the gradient‐induced phase must be inverted to maintain the intended interaction.

The phase accumulated by the gradients at spatial position r is as follows:

(A11)
$$\begin{array}{l} \phi_{\mathrm{grad}}\left(r,t\right)=\gamma \int_{0}^{t}G\left(\tau \right)\mathrm{rd}\tau =\gamma \int_{0}^{t}\left[G_{x}\left(\tau \right)x+G_{y}\left(\tau \right)y+G_{z}\left(\tau \right)z\right]d\tau \end{array}$$

Reordering the RF channels across the symmetry axis has already inverted the y‐dimension, effectively accounting for the inversion of $G_{y}$. To fully reverse the gradient‐induced phase, the remaining $G_{x}$ and $G_{y}$ gradient waveforms must also be inverted as follows:

(A12)
$$G_{x}\left(t\right)\to -G_{x}\left(t\right),y\to -y,G_{z}\left(t\right)\to -G_{z}\left(t\right)$$

We can apply the substitutions of Equation (A12) on Equation ([A11) to obtain the new phase accumulated by the following gradients:

(A13)
$$\begin{array}{l} \phi_{\mathrm{new}}\left(r^{\prime },t\right)=\gamma \int_{0}^{t}\left[-G_{x}\left(\tau \right)x-G_{y}\left(\tau \right)y-G_{z}\left(\tau \right)z\right]d\tau =-\phi_{\mathrm{grad}}\left(r,t\right) \end{array}$$

This inverted phase matches the original phase as introduced in Equation ([A11) but inverted. Therefore, the RF phase and the gradient‐induced phase align and preserve the intended excitation profile under the reversed $B_{0}$ direction.

### A.7. Conversion between gradient axis systems

Because the Siemens (source) system and Philips (target) system have differing axis system conventions for external pTx pulses that are decoupled from the FOV and subject orientation, we need to convert between those. Although documentation on these systems is relatively sparse, we have attempted to reconstruct these axis systems through cross‐verification of multiple sources and validation with MR experiments.

As illustrated in Figure A1, these axis systems only differ in direction, with all spatial directions flipped in sign—analogous to the difference between left‐handedness and right‐handedness:

- Philips: x is up, y is to the right, and z is out of the bore.
- Siemens: x is down, y is to the left, and z is into the bore.

To account for these differences and the $B_{0}$ conversion process, only the gradient waveform along the symmetry axis—which is already spatially inverted by reordering the RF channels—requires an additional explicit sign inversion. For the PASTeUR pulses used in this work, which are designed for a setup exhibiting symmetry in the $B_{1}^{\left(+\right)}$ sensitivities across the left–right dimension, the corresponding y‐axis gradient waveform must be inverted. The x and z gradients are already correctly inverted once and can therefore be mapped directly without further modification.
