# Supplementary material for: pTx‐Pulseq in hybrid sequences: Accessible and advanced hybrid open‐source MRI sequences on Philips scanners
Source: Magn Reson Med. 2025 Jul 3;94(5):1946–62. doi: 10.1002/mrm.30601 (PMC12393203; doi:10.1002/mrm.30601)
Supplement: Supplementary file 1 — Figure S1. Pulse sequence diagrams of a spiral sequence, resulting from spectrometer simulations while running the Pulseq interpreter. The sequence, sourced from the Pulseq GitHub repository, 20 is visualized using the vendor‐provided graphical viewer. Gradient waveforms are depicted in two coordinate systems: MPS (top) and XYZ (middle). [file MRM-94-1946-s001.html]

Supplementary Figure S1 

**Figure S1: Pulse sequence diagrams of a spiral sequence**, resulting from spectrometer simulations while running the Pulseq interpreter. The sequence, sourced from the Pulseq GitHub repository,20 is visualized using the vendor-provided graphical viewer. Gradient waveforms are depicted in two coordinate systems: MPS *(top)* and XYZ *(middle)*.
