# Supplementary material for: pTx‐Pulseq in hybrid sequences: Accessible and advanced hybrid open‐source MRI sequences on Philips scanners
Source: Magn Reson Med. 2025 Jul 3;94(5):1946–62. doi: 10.1002/mrm.30601 (PMC12393203; doi:10.1002/mrm.30601)
Supplement: Supplementary file 2 — Figure S2. Animation of real‐time average radiofrequency (RF) power monitoring during a parallel‐transmit pTx‐Pulseq scan, highlighting the dynamic control over the different transmit channels. The pTx‐Pulseq sequence ran during this animation consists of 20 two‐dimensional (2D) gradient‐echo (GRE) slices acquired with either one or two transmit channels active: first in increasing channel number, then decreasing, and finally the combination of first two sets. The active transmit channel is represented by an increase in the time‐averaged power, as shown in the power monitoring. This level will subsequently decrease when the channel is no longer active. [file MRM-94-1946-s002.html]

Supplementary Figure S2 

**Figure S2: Animation of real-time average RF power monitoring** during a parallel-transmit pTx-Pulseq scan, highlighting the dynamic control over the different transmit channels. The pTx-Pulseq sequence ran during this animation consists of 20 two-dimensional (2D) gradient-echo (GRE) slices acquired with either one or two transmit channels active: first in increasing channel number, then decreasing, and finally the combination of first two sets. The active transmit channel is represented by an increase in the time-averaged power, as shown in the power monitoring. This level will subsequently decrease when the channel is no longer active.
